# Personalized Medicine and Obstructive Sleep Apnea

**DOI:** 10.3390/jpm12122034

**Published:** 2022-12-08

**Authors:** Sy Duong-Quy, Hoang Nguyen-Huu, Dinh Hoang-Chau-Bao, Si Tran-Duc, Lien Nguyen-Thi-Hong, Thai Nguyen-Duy, Tram Tang-Thi-Thao, Chandat Phan, Khue Bui-Diem, Quan Vu-Tran-Thien, Thu Nguyen-Ngoc-Phuong, Vinh Nguyen-Nhu, Huong Le-Thi-Minh, Timothy Craig

**Affiliations:** 1 Sleep Lab Centre, Lam Dong Medical College, Dalat City 0263, Vietnam; 2 Immuno-Allergology Division, Hershey Medical Center, Penn State Medical College, Hershey, PA 15747, USA; 3 Sleep Lab Unit, Outpatient Department, Pham Ngoc Thach Medical University, Ho Chi Minh City 0028, Vietnam; 4 Department of Respiratory Functional Exploration, University Medical Center, University of Medicine and Pharmacy at Ho Chi Minh City, Ho Chi Minh City 0028, Vietnam; 5 Medical Education Center, University of Medicine and Pharmacy at Ho Chi Minh City, Ho Chi Minh City 0028, Vietnam; 6 Immuno-Allergology Department, Hai Phong Medical University, Hai Phong City 0225, Vietnam; 7 National Institute for Control of Vaccines and Biologicals, Ministry of Health, Hanoi City 0024, Vietnam; 8 Department of Physiology-Pathophysiology-Immunology, University of Medicine and Pharmacy at Ho Chi Minh City, Ho Chi Minh City 0028, Vietnam; 9 Pediatric Centre, Vinmec Times City International Hospital, Hanoi City 0024, Vietnam

**Keywords:** OSA, CPAP, apnea–hypopnea index, polysomnography, home sleep apnea testing, personalized management

## Abstract

Obstructive sleep apnea (OSA) is a common disease that is often under-diagnosed and under-treated in all ages. This is due to differences in morphology, diversity in clinical phenotypes, and differences in diagnosis and treatment of OSA in children and adults, even among individuals of the same age. Therefore, a personalized medicine approach to diagnosis and treatment of OSA is necessary for physicians in clinical practice. In children and adults without serious underlying medical conditions, polysomnography at sleep labs may be an inappropriate and inconvenient testing modality compared to home sleep apnea testing. In addition, the apnea–hypopnea index should not be considered as a single parameter for making treatment decisions. Thus, the treatment of OSA should be personalized and based on individual tolerance to sleep-quality-related parameters measured by the microarousal index, harmful effects of OSA on the cardiovascular system related to severe hypoxia, and patients’ comorbidities. The current treatment options for OSA include lifestyle modification, continuous positive airway pressure (CPAP) therapy, oral appliance, surgery, and other alternative treatments. CPAP therapy has been recommended as a cornerstone treatment for moderate-to-severe OSA in adults. However, not all patients can afford or tolerate CPAP therapy. This narrative review seeks to describe the current concepts and relevant approaches towards personalized management of patients with OSA, according to pathophysiology, cluster analysis of clinical characteristics, adequate combined therapy, and the consideration of patients’ expectations.

## 1. Introduction

Obstructive sleep apnea (OSA) is characterized by a partial or complete obstruction of the upper airways for at least 10 s, while exerting respiratory effort conducted by chest and abdomen movements. Symptoms of OSA include excessive daytime sleepiness, fatigue, snoring, repeated microarousals, and headache upon awakening. Diagnosis of OSA is based on clinical symptoms and polysomnography (PSG) at sleep lab or at home (home sleep apnea testing—HSAT). Treatment of OSA includes continuous positive airway pressure (CPAP), oral appliances, and surgery, in addition to weight loss and sleep hygiene.

However, OSA is a sleep disorder with high prevalence and comorbidities, and diverse clinical manifestations or phenotypes. Conventional approaches to diagnosis and treatment of OSA are no longer fully appropriate for all cases of OSA. To optimize the diagnosis and treatment of this disease, a more tailored approach is needed. The personalization (or individualization) in diagnosis and treatment of OSA is an optimal solution for all diseases in general and for OSA in particular. Within the framework of this article, the personalized approach to diagnosis and treatment of OSA will be discussed in each section. This personalized approach is based on the age group, common underlying diseases, and specific populations.

## 2. Personalization of Diagnosis and Treatment of Children with OSA

### 2.1. General Consideration

OSA in children is common. Although the prevalence of snoring in children is 3–12%, OSA affects 1% to 10% of children [1]. OSA affects the development of the child in terms of behavior, brain development, metabolism, and overall health. These harmful effects continue to affect children to adulthood. Therefore, the early diagnosis and appropriate treatment of OSA will help children to develop normally and to prevent future detrimental effects [2,3]. The most common age group with OSA is 2 to 8 years old, related to adenotonsillar hypertrophy which causes upper airway narrowing. Other causes, such as premature birth and Down syndrome, also increase the risk of OSA. Boys have a higher risk of OSA than girls after puberty, but before puberty it is equal in the two sexes [4]. Severity of OSA is increased when children are obese, exposed to cigarette smoke, or have family with difficult economic conditions [5,6].

### 2.2. Personalization of Clinical Approach for Children with OSA

#### 2.2.1. Overview

The history and symptoms of children with OSA are varied and complex. There are many validated questionnaires which have been used to screen children with OSA. However, the current treatment guidelines recommend that physicians should ask about sleep duration and quality of sleep, as well as snoring, in suspected children with OSA. Evaluation of the child’s sleep quality should include issues such as number of microarousals, abnormal sleeping positions, and sleep disruptions with unusual movements [4]. It is important to inform parents about sleep symptoms related to OSA because they may not perceive the problems as unusual or harmful to the child’s health [7].

Clinically, OSA in children often presents differently from adults with OSA. Children’s parents might discover OSA in their children with snoring, mouth breathing, pause of breathing, waking in the middle of the night, bedwetting, or enuresis. Children with OSA often experience sleep disruptions that affect daytime behaviors such as hyperactivity, inability to concentrate well, irritability, anger, aggression, or decreased memory. Parents of young children may pay more attention to their child’s daytime problems, and complain to the clinician about them, than their child’s nocturnal symptoms [8].

#### 2.2.2. Clinical Manifestations of Children with OSA

The symptoms of OSA in children vary by age group during childhood. From 3 to 12 months of age, newborn babies with OSA show signs of sleep disruption, poor day–night cycle discrimination, loud breathing or snoring, night sweats, poor feeding, physical retardation, ear infections, or persistent recurrent respiratory infections [1]. From 1 to 3 years of age, children with OSA often cry at night or panic during sleep, loudly breathing or snoring, mouth breathing, with short apnea, agitation at sleep, daytime fatigue, irritability, aggression, recurrent respiratory infections, or growth retardation [1]. Children from 3 to 6 years old have much more complicated and varied manifestations; in addition to the symptoms of younger children, they have additional symptoms such as abnormal sleeping positions, bedwetting, parasomnia, poor concentration, headache on waking, sleepiness during the day, or difficulty waking up in the morning [1]. School-age children with OSA usually show all the symptoms of younger children and others which are typical at this age, including loudly snoring, bruxism sleep, poor school study results, abnormal growth, delayed puberty, shyness and low self-esteem, depression, dental problems, and maxillofacial structure deformation [1,9].

In children, there are four main causes of OSA: obesity, adenotonsillar hypertrophy, abnormalities of craniofacial structure, and neuromuscular dysfunction. All of these cause narrowing and obstruction of the upper airway. Children with neuromuscular dysfunction may present with OSA and central sleep apnea (CSA). Structural craniofacial abnormalities are common in hereditary syndromes such as Crouzon, Pierre Robin, or Apert. Some other craniofacial abnormalities that cause short anterior pharynx space also increase the risk of OSA, such as chin hypoplasia, small chin or large tongue, and midfacial hypoplasia [4].

#### 2.2.3. Clinical and Laboratory Considerations for Children with OSA

Clinical examination of children with OSA may reveal excessive fatigue or hyperactivity. ENT (ear–nose–throat) examination may reveal allergic rhinitis with blocked nose, small chin, large tongue, narrow palate, or large tonsils. Similar to adults, obesity can be seen in children with OSA [8]. Blood pressure should be assessed for children with OSA because some children may develop hypertension due to prolonged OSA. Clinical examination also focuses on abnormal craniofacial structure with congenital syndromes. Physicians should ask carefully about a history of preterm birth as this is a risk factor for OSA [10].

Polysomnography (PSG) is the gold standard for the diagnosis of OSA in children and adults. Some other tests can support the diagnosis of OSA in children, such as respiratory polysomnography (RPG) or nocturnal oxygen saturation measurement, but the latter is not a substitute for PSG or RPG [4]. It is necessary to conduct more comprehensive tests for general consideration before developing specific treatments for OSA. These tests include electrocardiogram, chest X-ray, 24 h blood pressure monitoring, especially for children with severe OSA [11]. Imaging tests used to evaluate craniofacial anatomy are also necessary to contribute to the diagnosis and treatment of OSA in children [11].

All-night sleep polysomnography for children should be conducted with a full range of sensors on electroencephalogram, electro-optical, electromyography, electrocardiogram, respiratory exertion, nasal-to-mouth flow, nasal–oral temperature sensor, snoring, SpO2, and leg movements. The ideal case needs an additional CO_2_ sensor, which can be used either at the end of exhalation or under the skin. Measurement results need to be full of parameters of waking time, sleep quality, sleep potential, and sleep stages. The AHI (apnea–hypopnea index—the number of apnea events in 1 h) needs to be determined accurately. The OSA severity grade is as follows: 1/h ≤ AHI < 5/h mild; 5/h ≤ AHI < 10/h moderate; and 10/h ≤ severe AHI [4]. This classification only applies to children under 13 years old. Because the airway structure of infants is often highly stable and intact, the most common respiratory events are hypoventilation without apnea, as in adults. Hypoxia is also not as common as in adults and often, when determining respiratory events, an EEG will be needed to evaluate microarousal to identify hypoventilation related to microarousal. Therefore, if conducting respiratory polygraphs in children, the probability of missing diagnosis will be very high.

### 2.3. Personalized Treatment of Children with OSA

Treatment of OSA in children needs to follow a personalized approach within a general consideration. Weight loss is essential for obese children because it reduces the severity of OSA [12]. In obese children, the encouragement to physical activities is also very important: participating in sports may help to stabilize weight and improve the quality of sleep. Sleep hygiene is also important for healthy physiologic function and to reduce the risk of OSA.

Specific treatment for children with OSA includes surgical or non-surgical therapy. In OSA children with adenotonsillar hypertrophy and/or blocked nasal passages due to allergic rhinitis, the effectiveness of leukotriene receptor antagonists (LRAs) has been demonstrated for those with mild-to-moderate OSA [13]. LRAs might reduce the adenotonsillar size after 3 months of treatment and therefore improve the apnea–hypopnea index (AHI) results in children with OSA [14,15]. In cases of nasal problems, topical corticosteroid use might improve AHI in mild-to-moderate OSA [8]. The combination of LRAs and topical nasal corticosteroids may result in a significant reduction in AHI in children with OSA [12]. The recent systematic review and meta-analysis on the safety and adverse effects of intranasal corticosteroid therapy in children stated that the use of topical nasal corticosteroids which have been approved by Food and Drug Administration (FDA) are generally safe in the pediatric population: growth velocity reduction, hypothalamic–pituitary–adrenal axis suppression, and visual changes are uncommon [16].

For OSA children with adenotonsillar hypertrophy, adenotonsillectomy remains an optimal therapy for OSA. This intervention could be indicated for those with AHI ≥ 10 events/h or for those with mild to moderate OSA (5 < AHI < 10 events/h) but with severe symptoms [12,14,17,18]. Other interventions such as partial adenotonsillectomy or oropharyngeal orthopedic surgery have been ineffective and data are limited [12]. Tonsillectomy can be total or partial. The total tonsil and capsule are removed in total tonsillectomy; when the capsule or portion of the tonsil is left in situ this is known as intracapsular tonsillectomy [19]. However, when tonsillectomy fails, drug-induced sleep endoscopy (DISE) may provide a more personalized surgical plan and limit unsuccessful interventions [20].

**Figure 1:**
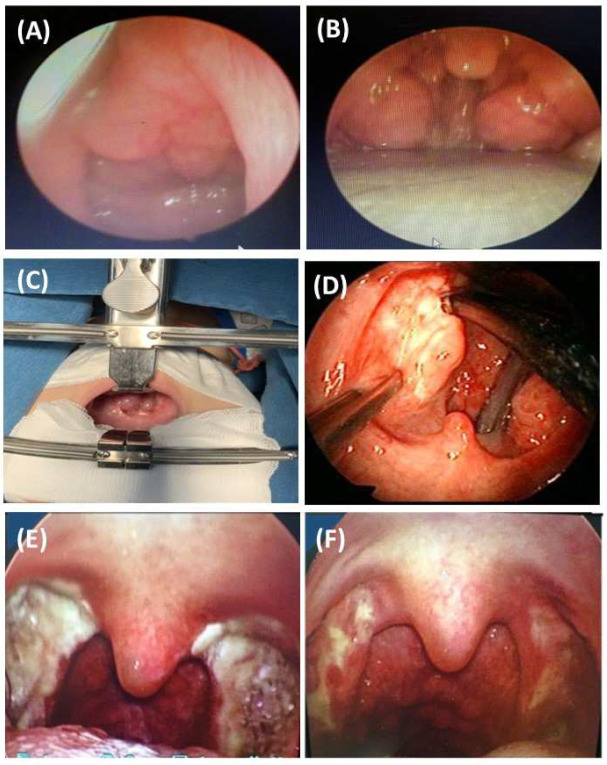
Tonsillectomy in children with OSA related to adenotonsillar hypertrophy. (**A**): adenoid hypertrophy; (**B**): tonsillar hypertrophy; (**C**,**D**): tonsillectomy; (**E**,**F**): the results of tonsillectomy at day 7 and 14 after intervention.

Children should have a careful follow-up after tonsillectomy. Regarding surgical intervention for adenotonsillar hypertrophy, children with obesity and allergic rhinitis must have those health issues addressed before surgery. Adenotonsillectomy is effective in reducing AHI in children who are not obese and without nasopharyngeal problems or other causes [21]. The results of our recent publication, based on a series of 114 children (mean age of 5.5 ± 2.1 years) with OSA who had adenotonsillar hypertrophy, demonstrated that treatment with ALR for moderate OSA or surgery (Figure 1) for severe OSA might reduce the symptoms related to OSA at night and during the day, and the mean AHI post-intervention [22].

The same as with adults, the use of positive airway pressure (PAP) is also an option for the treatment of pediatric OSA. Although there are many limitations for the treatment of PAP in children with OSA, PAP may be indicated in cases of moderate-to-severe OSA in obese children awaiting weight loss, or in children who are indicated for adenotonsillectomy that has not yet been performed [8]. However, adherence to PAP therapy in children is a big concern because ensuring their cooperation is more difficult than for adults. In addition, the long-term use of nasal or face masks can also affect the development of the child’s craniofacial structure. Oral devices in children do not have much data to evaluate the effectiveness and benefits. For children with a narrow and high palate, palatal expander intervention might improve AHI [23]. Maxillomandibular advancement surgery might be considered a primary single-stage treatment for improving the quality of life in preadolescent refractory OSA [24]. In addition, the efficacy of orthodontic treatment versus adenotonsillectomy, in children with moderate OSA accompanied by tonsillar adenoid hypertrophy and mandibular retrognathia, has been demonstrated recently [25].

However, when tonsillectomy and adenoidectomy is not indicated or fails to address pediatric OSA (residual pediatric OSA), the orthodontic interventions might be considered as alternative methods for those with maxillary expansion, mandibular advancement, or maxillary complex advancement with skeletal anchored headgear [26].

## 3. Personalization of Clinical Approaches for Adults with OSA

### 3.1. Overview

The conventional diagnostic and therapeutic approaches for adults with OSA are usually based on clinical assessments, followed by polysomnography (PSG) or respiratory polygraphy (RPG) in a sleep lab or at home (HSAT: home sleep apnea testing), and treatment decisions. With this approach, it could be unadvisable for physicians to decide the treatment approach for OSA without evaluating AHI. CPAP therapy is an optimal treatment for adults with severe OSA, as it has been demonstrated previously [27,28,29,30]. However, there are some patients who do not tolerate PAP therapy. Patients with OSA-associated asthma or COPD as comorbidities (OLDOSA: obstructive lung disease and OSA) should have a personalized approach to devise an optimal treatment plan.

### 3.2. Personalization of OSA with Phenotype Approach

The phenotypic stepwise approach is one of the strategies to optimize treatment efficacy for patients with OSA. The definition of OSA phenotypes is described as the combination of disease features and clinical relevance of OSA (symptoms, response to treatment, health status, and quality of life) [31]. Based on the clinical symptoms of each individual patient, physicians must have a personalized approach. Depending on the personalization of phenotypic characteristics of patients, the response to treatment of each individual patient is also different. Adherence to CPAP therapy might be more difficult with greater possibility of failure in patients with OSA complicated by anxiety disorders or depression. Although there are not enough data reported on symptom-based phenotypes to achieve a high therapeutic effect, the personalization of OSA management in adults should be performed, and based on the specific symptoms and populations.

The personalization of OSA management based on AHI may be considered as a corner stone for phenotype-based approaches, although AHI has an inaccurate reflection of OSA severity between men and women [32]. However, when analyzing the PSG to determine its correlation with cardiovascular morbidity and mortality, severe nadir hypoxemia might increase the risk of cardiovascular disease and death in patients with similar AHI. In addition, periodic leg movements have also been found to be associated with an increased risk of cardiovascular disease and death. Some studies have shown that the degree of hypoxia and the duration of hypoxia [33], or the duration of respiratory events [34] can replace the AHI when assessing the degree of OSA severity.

The personalization of OSA management based on the phenotype treatment response is also an approach that helps to achieve good efficacy. Prescribing CPAP therapy is the optimal treatment option for adult patients with severe or moderate symptomatic OSA because the majority of OSA patients tolerate this treatment well. Therefore, CPAP therapy focus on additional objectives such as lowering blood pressure, stabilizing blood glucose, and improving life expectancy [35]. There are a few patients with OSA who still have residual AHI despite optimal titration of CPAP pressure. This may be due to the development of central apnea after PAP treatment.

The personalization of OSA management needs more data on specific clinical and functional features of OSA to refine the description of OSA phenotypes. This would require the connection of multiple sleep data sources for the purpose of analyzing and synthesizing OSA-specific phenotypes.

### 3.3. Personalization of OSA with Function and Pathophysiological Approach

In its physiopathology, OSA is mainly due to the anatomical abnormalities of the laryngo-pharynx, inducing the narrow or complete obstruction of the upper airway during sleep. However, besides the anatomical factors, there are also non-anatomical factors that contribute to OSA severity and complicate the treatment of OSA, including oversensitivity to the ventilation control system, low threshold for respiratory stimulation, and poor tonus and responsiveness of pharyngeal muscles while sleeping [1,2,3]. These factors will influence the obstruction of the upper airway in OSA and also determine the tolerability, adherence, and detrimental response to the treatment with PAP [36,37,38].

The personalization of OSA management may help then to differentiate between OSA patients due to anatomical structure and those not due to anatomical abnormality. Thus, it might be unreasonable to grade the severity of OSA based on AHI alone because it limits the effectiveness of optimal therapy in patients with OSA [39].

For OSA patients with upper airway obstruction, in more than 50% of them, it is anatomically multi-segmental and would require multi-level surgical intervention [40]. However, previous studies have advocated that multi-level surgery regarding the soft palate and the base of the tongue is safe and successful [41,42]. The use of DISE can demonstrate the level of obstruction at the base of the tongue and/or the epiglottis, and can predict the outcome of upper airway surgery [43,44]. With single-level palatal surgery, patient selection with DISE has attained better long-term outcomes [45]. Some suggestions for the personalized treatment of patients with OSA not due to anatomical abnormalities are: (1) patients with OSA and with low loop gain (ventilatory response/ventilatory disturbance < 1): an oral appliance can be used or upper airway surgery can be performed [46,47,48]; (2) patients with high loop gain (ventilatory response/ventilatory disturbance > 1): respond to supplemental oxygen and are considered “responders” if their AHI is reduced by ≥50% with oxygen therapy and otherwise considered “non-responders” [49,50,51]; (3) in patients with weak oropharyngeal muscle tone, stimulants can be used to increase upper respiratory muscle tone [52]. The role of myofunctional therapy in the treatment of OSA has been also emphasized in the recent recommendations as an adjunct in the management of OSA. These recommendations suggest that this treatment modality, consisting of exercises targeting oral and oropharyngeal structures, can lead to a reduction in the AHI and snoring, and can improve oxygenation during sleep and daytime sleepiness [53,54,55].

However, the identification of the non-structural OSA patients remains a challenge because it requires more advanced techniques such as magnetic resonance imaging (MRI) or the use of esophageal sensors in combination with CPAP therapy to assess upper airway narrowness. These techniques are invasive and expensive [37,56,57]. Therefore, it is necessary to differentiate patients who could use conventional and more readily available tests, such as PSG, RPG, or other non-invasive sleep monitoring devices which are non-invasive and easy to perform, from those who need more advanced forms of evaluation.

In the future, personalized medicine in OSA should rely on data from randomized controlled studies, and focus on the pathophysiological mechanisms and clinical phenotypes of patients with OSA. These multidirectional approaches also help to find out accurate therapies and relevant solutions to treat patients with OSA or to re-evaluate OSA patients with treatment failures. Therefore, the future diagnosis and treatment of OSA should coordinate these multidirectional approaches to solve the individual patient’s problems related to OSA in long-term follow-up.

## 4. Personalization of Clinical Approaches for OSA in Elderly Patients

### 4.1. General Considerations

OSA is a common disorder in older people. Population-based studies have shown that the prevalence of OSA increases with age. Overall, the prevalence of people over 65 years old having sleep apnea is between 13 and 32% [58]. A 10-year age augmentation is associated with an increase in the odds of having an AHI of 15 or greater, by 24% [59]. Male gender and obesity are not important risk factors for OSA after the age of 50 years old. The male-to-female ratio for OSA in the elderly is 1:1.

The pathological mechanisms underlying OSA change with age. Aging is associated with an increase in pharyngeal collapsibility and in pharyngeal resistance during sleep, independent of body mass index (BMI) and gender [60]. A decrease in the activity of the dilator muscles of the pharynx by the loss of tissue elasticity may contribute to pharyngeal collapse [61]. The response of the genioglossus muscle to negative intrapharyngeal pressure during sleep is reduced [62]. For older women, decreased levels of sex hormone may partly be responsible for increased collapsibility of the posterior oropharynx [63].

### 4.2. Personalization of OSA with Clinical Approach in Elderly Patients

The clinical characteristics of OSA in older adults are probably different from those observed in younger ones. While the classic symptoms of OSA like apneas, nocturnal choking, and daytime sleepiness are witnessed, older patients are more likely to present with sleep complaints, nocturia, and cognitive dysfunction [58,64]. Snoring may be less present or unrecognized in older people with OSA [59]. Excessive daytime sleepiness (EDS) in older patients is less severe than in younger subjects with the same level of OSA severity. However, it may be difficult to evaluate sleepiness in older adults, because the Epworth Sleepiness Scale (ESS) is not a validated tool in this age group. There was evidence that ESS might underestimate sleepiness severity in older adults [65]. Moreover, EDS in the elderly is highly influenced by pre-existing neurocognitive decline, comorbidities, and the use of medication.

Growing evidence suggests an increased risk of cognitive impairment in older adults with OSA. However, the results are heterogeneous because of differences in OSA definitions, types of neuropsychological tests, and variables adjusted for in statistical analyses. Attention, vigilance, memory, and executive function are the cognitive domains most commonly affected in OSA patients [66]. Several factors associated with cognitive dysfunction are snoring, apneas, nocturnal hypoxemia, and AHI [67]. There are hypothetical mechanisms which may explain the association between OSA and cognitive dysfunction. Repeated micro-arousals which happen during the sleep of OSA patients alter both sleep macro-architecture (time in stage 3 or deep sleep and REM sleep) and micro-structure (K-complexes, spindle and slow wave characteristics) [66,67]. These factors have been proven to play important roles in neurogenesis, synaptic plasticity, memory formation, and consolidation [68]. Therefore, chronic sleep alterations in OSA can negatively affect not only the cognitive functions but also changes in brain structure.

OSA has been known to be a risk factor of cerebro-cardiovascular diseases, including hypertension, congestive heart failure, stroke, arrhythmias, ischemic events, and pulmonary artery hypertension. However, these relationships are less evident in the elderly population. The cardiovascular risk was more likely to increase in younger (<65 years old) than older subjects in the Sleep Heart Health Study [69]. Another prospective observational study showed that older patients (≥65 years old) with untreated severe OSA (AHI > 30/h) had increased all-cause and cardiovascular mortality [15]. In one population-based cohort study in older patients (mean age 77 years), severe OSA (AHI ≥ 30/h) increased the risk of ischemic stroke, independently of known risk factors [70].

In addition, OSA in elderly patients is associated with multiple potential consequences, including an increased risk of falls in older men, depression, and decreased quality of life, because of reduced social functioning and vitality [71,72].

### 4.3. Personalized Treatment for OSA in Elderly Patients

Regarding the treatment of older adults with OSA, positional measures and oral appliances are recommended in mild-to-moderate OSA [64]. Positive airway therapy or CPAP is the treatment of choice in patients with the moderate-to-severe or symptomatic form of OSA. There is not much evidence on the effectiveness of OSA with CPAP in this age group. A few studies have shown that CPAP may reduce daytime sleepiness, cardiovascular consequences, and cognitive outcomes [73]. Concerning the patients’ compliance with CPAP, 30% of the elderly with OSA refuse it, and only about 41% remain adherent to their treatment after one year. In patients over 80 years old, the compliance with CPAP is very low (less than 3 h per night) [73]. Adherence to CPAP therapy in older patients may be affected by factors such as cognitive impairment, medical and mood disturbances, nocturia, and lack of a supportive partner [64]. Behavioral interventions can improve CPAP adherence in the elderly.

OSA is prevalent but underdiagnosed in older patients for many reasons, such as non-specific symptoms, comorbidities, polypharmacy, or the social belief of sleep problems as normal aging. The clinical presentation of OSA in older adults differs from that of younger patients. Female gender and obesity are not important risk factors in older adults. Polysomnography is the gold standard in the diagnosis of OSA in elderly. Respiratory polygraphy could be used in patients with comorbidities or neurocognitive decline. Moderate-to-severe OSA is associated with increased risk of cardiovascular diseases as well as cognitive impairment, and CPAP treatment may reduce the risk. However, compliance with CPAP in older patients is low. Considering the high prevalence and correlation with medical and mental comorbidities, health care practitioners should incorporate OSA screening in clinical practice.

## 5. Personalization of Management for Patients with OSA and Comorbidities

### 5.1. Background

Personalized medicine in OSA should focus on the management of patients’ comorbidities. Comorbidities of OSA are more common in adult or elderly patients than children. These include chronic respiratory diseases, cardiovascular diseases, and endocrinological or neurological disorders. The optimal management of OSA, using a personalized approach, should target comorbidities which may improve patient outcomes. This section focuses only on the common comorbidities in patients with OSA.

### 5.2. Personalized Approaches for Patients with OSA and Airway Diseases

#### 5.2.1. OSA and Allergic Rhinitis

The first upper airway disease which is comorbid with OSA is allergic rhinitis (AR), called AROSA (allergic rhinitis and OSA) [74]. A meta-analysis of 44 studies involving 6086 participants found that the proportion of OSA adults with AR was 35.2% (95% CI, 25.6–44.7) and the percentage of OSA children with AR was 45.2% (95% CI, 25.4–65.0) [75]. The prevalence of OSA in AR patients may even be up to 79.7% in young people [76]. The results of our previous study showed that OSA in patients with persistent allergic rhinitis was more severe than in healthy subjects (AHI = 17 ± 12 vs. 6 ± 3; *p* < 0.01); in particular, treatment with an antihistamine combined with a leukotriene receptor antagonist, or with intranasal steroid alone, made a significant reduction in the severity of OSA, measured by AHI (8 ± 4 vs 17 ± 12; *p* < 0.01) [77].

**Figure 2:**
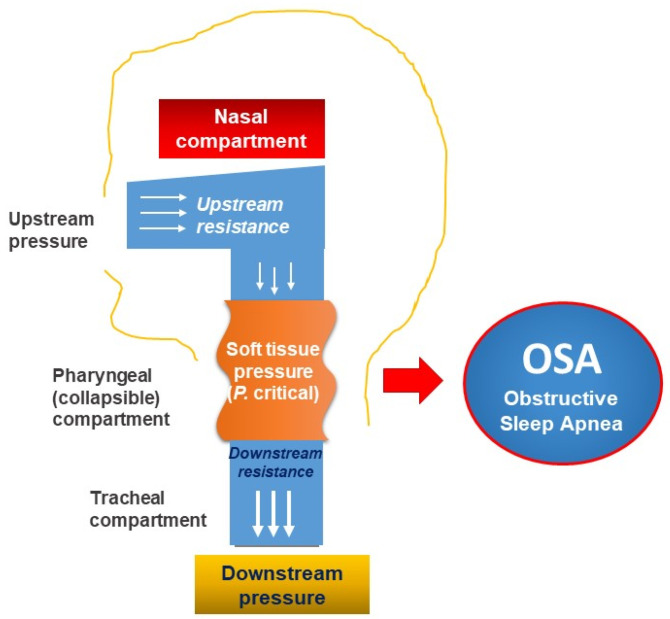
Mechanism of obstructive sleep apnea (OSA) related to pharynx collapse due to the increase of upstream and downstream pressure and resistance.

Thus, personalized approaches for patients with AROSA should be based on the pathophysiology of both diseases. The pathogenesis of the association between AR and OSA (AROSA) is quite complex. The nose and upper airway regulate more than 50% of airway resistance, which plays a very important role in performing the physiological function of the respiratory system. Frequent unilateral or bilateral nasal congestion results in a significant increase in total airway resistance [78]. In addition, according to the Starling resistance model, the upper airway acts as a hollow tube which is capable of constricting proximal of the inlet (nostril) and in the posterior segment of the collapse of the pharynx; the presence of an obstruction upstream (nose) will create a negative pressure (suction) in the downstream (pharynx), leading to collapse of the pharynx (soft tissue structure) in “at-risk individuals” (Figure 2). Moreover, the increased resistance in the nose will increase the habit of breathing through the mouth, making the upper airway unstable (easy to collapse) and inducing OSA [79,80].

The mediators produced from AR, such as histamine, cysteinyl leukotrienes, and interleukins, also cause the manifestations of nasal obstruction and sleep disruption in patients with OSA. Patients with OSA also have elevated levels of mediators such as tumor necrosis factor TNF, interleukin 6, and interleukin 1, which are Th2-activating cytokines, which may aggravate symptoms of AR [81]. Thus, the personalized management of AROSA should be based on the use of topical corticosteroids and leukotriene receptor antagonists [75,81]. The treatment of AR with intranasal corticosteroids may reduce the symptoms of the disease, especially nasal congestion. This personalized treatment combined with improved sleep hygiene or weight loss may improve fatigue, excessive daytime sleepiness, and quality of life in patients with AROSA [75].

#### 5.2.2. OSA and Obstructive Lung Diseases

**Figure 3:**
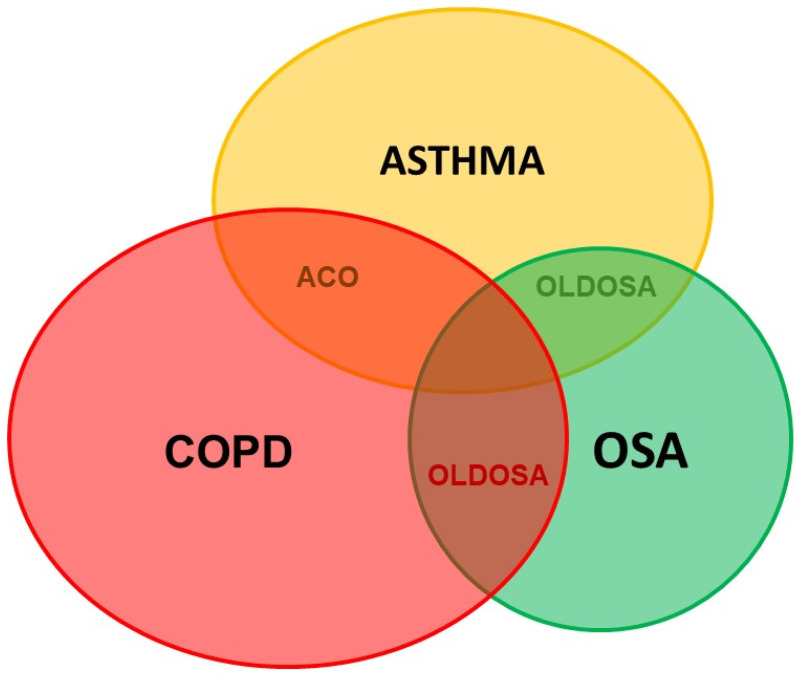
Comorbid obstructive lung disease and obstructive sleep apnea. ACO: Asthma–COPD overlap; COPD: chronic obstructive lung disease; OSA: obstructive sleep apnea; OLDOSA: obstructive lung disease and obstructive sleep apnea.

Obstructive lung diseases (OLDs) such as COPD (chronic obstructive pulmonary disease) and asthma are usually comorbid with OSA and named OLDOSA (obstructive lung disease and OSA) (Figure 3). OLDs share common risk factors with OSA such as smoking, obesity, and GERD [82,83,84]. In a 10-year longitudinal follow-up study of 4980 patients with asthma, COPD, and OSA as comorbidities, the 10-year cumulative all-cause mortality was 52.8%; median time to death was 2.7 years. Rate of death in the comorbid group was: COPD–OSA 53.2%, asthma–COPD 62.1%, asthma–OSA 63.5%, and asthma–COPD–OSA 67.8% [85]. In our multicenter study published previously, the percentage of patients having moderate or severe OSA, defined as AHI > 15/h, was significantly higher in subjects with asthma–COPD overlap (ACO) than that in subjects with asthma and COPD (64.4% versus 35.5% and 36.4%; *p* < 0.01 and *p* < 0.01, respectively). In addition, the mean AHI in patients with ACO was significantly higher than that in those with asthma and COPD (*p* < 0.05 and *p* < 0.05, respectively) [84].

OSA and OLD have a bidirectional effect probably as a result of the common risk factors, nasopharyngeal pathology, increased airway resistance, hypoxemia, bronchospasm, local and systemic inflammation, and anti-inflammatory therapy [82]. All of these conditions have nocturnal hypoxemia, but OSA is characterized by intermittent hypoxemia during the night, while in COPD or asthma hypoxemia is continuous, both day and night, and is often aggravated at night. To diagnose OSA in patients with OLD, the attended PSG in sleep lab should be performed.

The management of patients with OLDOSA should be personalized. Treatment with CPAP in patients with OLDOSA may reduce mortality and improve quality of life compared with no CPAP [86]. CPAP improves day and night symptoms in asthmatic patients with OSA, reduces bronchodilator use and exacerbations, and improves lung function and quality of life. Treatment with CPAP in patients with OLDOSA needs to be personalized to improve patient adherence. CPAP treatment in adults and tonsillectomy in children with OLDOSA should be done only after optimizing the treatment of OLD. Due to the high prevalence of OSA in OLD, OSA should be screened in patients with OLD in order to confirm OLDOSA, personalize the therapy, and improve quality of life and mortality in these patients.

### 5.3. Personalized Approaches for Patients with OSA and Cardiovascular Diseases

#### 5.3.1. General Considerations

The personalized management of OSA in patients with cardiovascular diseases is very important because the prevalence of comorbid cardiovascular diseases and OSA (CAVADOSA) is very high [87,88]. CAVADOSA has a negative impact on patient outcomes due to the increase in fatal and non-fatal cardiovascular morbidity and mortality. CAVADOSA may also reduce the patient’s quality of life and increase medical costs due to the severity of OSA [87]. Therefore, the personalization of CAVADOSA management is based on the early diagnosis and the appropriate treatment of cardiovascular diseases with comorbid OSA to effectively improve patient outcomes [89].

#### 5.3.2. Personalization of OSA Diagnosis in Patients with Cardiovascular Diseases

Despite the high frequency of CAVADOSA, it is often underdiagnosed. This may be due to the lack of interest from cardiologists or the lack of diagnostic facilities such as PSG or RPG and available sleep labs. Furthermore, patients with CAVADOSA rarely complain about their sleep quality and snoring to cardiologists. In addition, a significant proportion of patients with CAVADOSA do not present with common characteristics of an OSA patient, such as male gender, daytime sleepiness, or snoring. In patients with CAVADOSA, the predominant symptoms are often related to the underlying cardiovascular disease [90,91]. Therefore, to diagnose and treat OSA early in cardiovascular patients, a personalized approach will help physicians to actively identify high-risk patients for CAVADOSA with resistant hypertension, recurrent atrial fibrillation, or nocturnal angina. Appropriate investigations such as sleep medicine consultation within attended PSG in available sleep labs should be used to further evaluate these patients.

#### 5.3.3. Personalization of OSA Treatment in Patients with Cardiovascular Diseases

In patients with CAVADOSA, both conditions should be treated concurrently. Lifestyle modifications are important for CAVADOSA. The role of ideal body weight maintenance has been confirmed in these patients [92]. Interestingly, 10% of weight gain may increase the risk of OSA by six times [93]; inversely, 10% of weight loss might reduce the severity of AHI by 26% [94]. The personalized treatment of OSA in subjects with CAVADOSA should also focus on alcohol cessation and smoking cessation which would simultaneously reduce the risk of cardiovascular disease.

Among the specific treatments for OSA in patients with CAVADOSA, PAP (positive airway pressure) with CPAP, BiPAP (bilevel positive airway pressure), or auto SERVO should be personalized to achieve optimal outcomes. Although CPAP therapy is the most studied treatment in patients with CAVADOSA, the alternative methods should be individualized in these patients. Adequate treatment with CPAP might reduce major cardiovascular and cerebrovascular events, risk of stroke, transient ischemic accident, and fatal and nonfatal cerebrovascular events [87]. The personalized treatment of CPAP for patients with CAVADOSA should be prioritized as current recommendations suggest the use of CPAP at least four hours per night and five days per week [87]. Measures to improve CPAP adherence and effectiveness should be personalized for each patient with CAVADOSA.

### 5.4. Personalized Approaches for Patients with OSA and Diabetes

#### 5.4.1. General Considerations and Personalized Approach

Type 2 diabetes mellitus (T2DM) and OSA are closely related and comorbid with significant public health implications. Both diseases have a high incidence and overlap in common risk factors, including obesity, older age, and higher prevalence in men [95]. OSA is an independent risk factor for the development of T2DM and the prevalence of T2DM in OSA patients is between 15–30%. In addition, severe OSA also leads to poorer glycemic control in T2DM patients [96]. The meta-analysis conducted by Huang et al. demonstrated that the risk of developing OSA was 2.14-fold (95% CI, 1.49, 3.07) in patients with T2DM after adjusting for age; the risk of T2DM after 10–18 years in OSA patients was 2.97-fold (95% CI, 2.40, 3.69) and decreased to 2.06 (1.86, 2.28) after adjusting for multiple confounding factors [95]. The prevalence of OSA in obese T2DM is up to 86.6% [96].

#### 5.4.2. Personalized Treatment of OSA Patients with Comorbid Diabetes and Metabolic Syndrome

OSA has also been shown to be closely comorbid with metabolic syndrome. The term “Z syndrome” has been used to describe the association between obesity, insulin resistance, hypertension, and dyslipidemia with OSA. The OR (odds ratio) index of metabolic syndrome in OSA patients ranges from 5 to 9-fold when compared with subjects without OSA, and independent of age and BMI [97,98,99]. Due to the bidirectional association between T2DM or metabolic syndrome and OSA [75,96,100,101,102], the personalized management of these comorbid diseases should be performed to avoid the high risk of fatal cardiovascular events.

In severe or symptomatic OSA patients, CPAP therapy is the main approach because of its effectiveness in reducing AHI and symptoms. Although several trials have shown that CPAP therapy in OSA patients with T2DM did not significantly reduce HbA1C levels or BMI, nor improve blood glucose, CPAP therapy could improve insulin sensitivity and reduce insulin resistance assessed by the HOMA-IR (Homeostatic Model Assessment Index) [103,104,105]. However, the common limitation of most trials is that the duration of CPAP used during sleep is usually less than 4 h. In another study [106] patients who took CPAP for at least 4 h/night (6.6 h/night) could achieve a significant improvement in HbA1C. The personalization of OSA management in patients with comorbid T2DM should be based on weight loss (for overweight and obese people) for helping to better control blood sugar and reducing the AHI; in particular, 10% of weight loss can predict a 26% decrease in AHI [94]. More recently, randomized controlled trials with 4 years of follow-up have indicated that weight loss can alter OSA severity with a mean AHI reduction of 0.78 events/h for every kilogram of weight lost [107,108].

### 5.5. Personalized Approaches for Patients with OSA and Insomnia

#### 5.5.1. Personalized Diagnosis

A typical symptom of OSA is hypersomnia which is defined by daytime sleepiness. However, some patients with OSA have the symptoms of insomnia, named COMISA (comorbid insomnia and OSA). Due the high prevalence of insomnia and COMISA in the general population and in patients with OSA [109,110], and due to the complexity in diagnosis and treatment of COMISA, the management of this disease should be personalized. Insomnia is defined as difficulty falling asleep, waking up in the middle of the night and having difficulty getting back to sleep, or waking up earlier than desired with an impact on daytime functioning [111]. Patients with COMISA often have more severe daytime symptoms, and the frequency of neuropsychiatric and cardiovascular disorders is also higher than in the general population. A previous study showed that, among patients with COMISA, 35% had AHI ≥ 5 times/hour and 29% had AHI ≥ 15 times/hour [112].

#### 5.5.2. Personalized Treatment

For patients with COMISA, personalized treatment aims to resolve different challenges including sedative-induced severe OSA, CPAP treatment-induced insomnia, poor CPAP adherence, and the efficiency of balanced OSA—insomnia treatment. Luyster et al. reported on symptoms of OSA and insomnia and showed that only a few clinical features were distinct, with most daytime and nocturnal complaints shared by both patients; and there was a significant overlap in symptoms between insomnia and OSA (COMISA). Hence, it is difficult to determine a causal relationship between OSA and insomnia in individual patients. Different treatment responses may indicate whether OSA is primary or secondary to the insomnia component and vice versa.

The personalized diagnosis and treatment of COMISA always requires multidisciplinary collaboration. Combined therapy, including cognitive behavior therapy (CBT) for the treatment of insomnia and for OSA, may result in a greater improvement than treatment with CPAP alone [110]. The assessment of symptom improvement in patients with COMISA should be measured by the degree of insomnia and daytime function. In these patients, pre-administered CBT for insomnia might increase CPAP tolerance and improve insomnia in comparison with CPAP alone. The effectiveness of CBT remains unaffected by OSA.

### 5.6. Personalized Approaches for Subjects with OSA and Genetic Defects and Other Disorders

#### 5.6.1. Personalized Approach and Diagnosis

The personalized approach in patients with genetic defects and OSA is necessary for optimizing the patients’ outcomes. Particularly in individuals with Down syndrome (DS), OSA is observed in up to 85–93% compared with 7 to 13% of the general population [113,114]. The prevalence of OSA in these subjects is associated with a variety of genetic, anatomical, endocrine, and metabolic abnormalities. Its main cause is an extra chromosome at position 21 causing asymmetrical skull structure, upper airway narrowing, hypotonia, hypothyroidism, or obesity [115,116,117]. Both children and adults with DS are particularly affected by OSA.

The personalized diagnosis of OSA in patients with DS (DOSOSA: Down syndrome and OSA) is based on the clinical characteristics and PSG. Neurophysiological analysis should be performed to identify the specific features of PSG. In the DS population, PSGs usually report a high prevalence of OSA and an association between degree of mental retardation and duration of REM sleep, which might play a potential role in deficit perception of people with DS [118,119]. Sleep fragmentation caused by apnea/hypopnea episodes might be considered as the cause of neurocognitive dysfunction, impaired quality of life, and increased risk of occupational accidents in patients with DOSOSA. In the DS population, obesity is very common and develops from childhood. The relationship between obesity, fat deposition, and OSA severity has been well established in the DOSOSA population [105,120,121,122]. Obesity is a pathology more frequent in people with DS because they have the genetic predisposition to develop obesity [123].

Moreover, there seems to be a link between genetic factors and the development of OSA-related cognitive decline. The ε4 isoform of apolipoprotein ε (Apoε4) has been found to be an early marker of Alzheimer’s disease [124]. It is overexpressed in subjects with DS, confirming the risk of developing this disease. Thus, OSA-related cognitive disorders and the development of Alzheimer’s disease are also closely linked [125]. Other genetic factors may also influence craniofacial morphology and obesity [126,127], and thus may increase the risk of OSA [128,129].

#### 5.6.2. Personalized Treatment

Currently, there is no personalized and potential treatments for OSA in subjects with genetic defects. CPAP therapy may be an ineffective treatment option because of poor tolerability and adherence [130]. Therefore, the personized approach should focus on the necessity of early diagnosis of OSA in this population to minimize its harmful complications. The current personalized and alternative therapy without CPAP in people with DOSOSA with non-severe AHI is based on a program of physical activity combined with the implementation of rigorous dietary and lifestyle measures [131].

**Figure 4:**
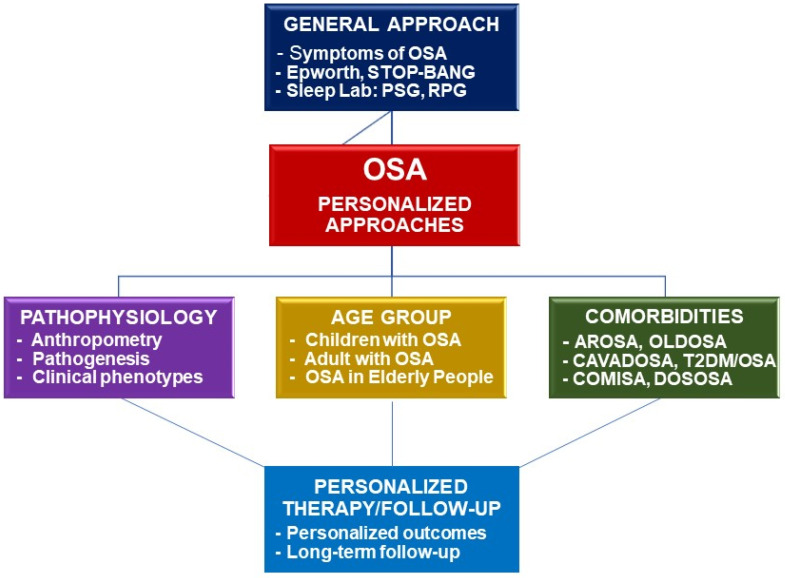
Framework of personalized approaches for diagnosis and treatment of OSA. OSA: obstructive sleep apnea; PSG: polysomnography; RPG: respiratory polygraphy; AROSA: allergic rhinitis and OSA; OLDOSA: obstructive lung disease and OSA; CAVADOSA: cardiovascular diseases and OSA; T2DM: type 2 diabetes mellitus; COMISA: comorbid insomnia and OSA; DOSOSA: Down syndrome and OSA.

Finally, the diagnosis and treatment of OSA comorbid with other disorders should be personalized for having an accurate diagnosis and optimal treatment for each given patient in clinical practice. For each given comorbid disease, the personalized patient with OSA should benefit from a multidimensional approach and treatment (Figure 4).

## 6. Conclusions

The personalization of approaches to diagnosis and treatment of OSA is necessary to improve the patients’ outcomes. Personalized medicine in OSA is a new strategical approach because the old methods could not effectively manage all the crucial issues in diagnosis and treatment of OSA patients. The personalized approach works to respond adequately to the specific requirements of each individual patient to achieve appropriate and optimal treatment. Although there has been much progress in diagnosis and treatment of OSA in the last few decades, the personalized and comprehensive approach will lead to more effective management for patients with OSA.

## Data Availability

Not applicable.
